# Prognostic Epstein-Barr Virus (EBV) miRNA biomarkers for survival outcome in EBV-associated epithelial malignancies: Systematic review and meta-analysis

**DOI:** 10.1371/journal.pone.0266893

**Published:** 2022-04-18

**Authors:** Mai Abdel Haleem Abusalah, Ahmad Adebayo Irekeola, Rafidah Hanim Shueb, Mu’taman Jarrar, Chan Yean Yean

**Affiliations:** 1 Department of Medical Microbiology and Parasitology, School of Medical Sciences, Universiti SainsMalaysia, Kelantan, Malaysia; 2 Microbiology Unit, Department of Biological Sciences, College of Natural and Applied Sciences, Summit University Offa, Offa, Kwara State, Nigeria; 3 College of Medicine, Imam Abdulrahman Bin Faisal University, Dammam, Saudi Arabia; University of Sussex, UNITED KINGDOM

## Abstract

**Background:**

The EBV-associated epithelial tumours consist 80% of all EBV-associated cancer, where the nasopharyngeal cancer (NPC) and EBV-associated gastric carcinoma (EBVaGC) are considered as the most frequent EBV-associated epithelial tumours. It has been shown that the BART-encoded miRNAs are abundantly expressed in EBV-associated epithelial tumours, hence, these miRNAs may serve as diagnostic and prognostic biomarkers for EBV-associated epithelial tumours. Therefore, the purpose of this systematic review and meta-analysis is to assess these EBV miRNAs as prognostic biomarkers for NPC and GC.

**Method:**

This systematic review was developed based on PRISMA guidelines and utilizing PubMed, Web of Science, Scopus, Cochrane, and Google scholar databases. The retrieved articles were thoroughly screened in accordance with the selection criteria. The hazard ratio (HR) and 95% confidence interval (CI) for patient survival outcomes were used to evaluate EBV miRNA expression levels. To assess the risk of bias, funnel plot symmetry and Egger’s bias test were employed.

**Result:**

Eleven studies met the selection criteria for inclusion, and four were included in the meta-analysis. Most of the articles considered in this study were from China, with one study from South Korea. The overall pooled effect size estimation (HR) for upregulated EBV miRNAs was 3.168 (95% CI: 2.020–4.969), demonstrating that upregulated EBV miRNA expression enhanced the mortality risk in NPC and GC patients by three times.

**Conclusion:**

To the best of our knowledge, this is the first meta-analysis that investigates the significance of EBV miRNAs as prognostic biomarkers in NPC and GC patients. The pooled effect estimates of HR of the various studies revealed that higher EBV miRNA expression in NPC and GC may result in a worse survival outcome. To assess the clinical significance of EBV miRNAs as prognostic biomarkers, larger-scale prospective studies are needed.

## Introduction

The Epstein-Barr virus (EBV) is a lymphotropic herpesvirus belonging to the human gamma herpesvirus group. EBV infects >95% of the global individuals throughout childhood and early adolescence, and the virus does not cause any symptoms [1]. Despite its strong relationship with a variety of epithelial and lymphoid cancers, the virus does not cause significant symptoms in most lifelong carriers who have EBV-infected B memory cells [2–4]. Infection with EBV is associated with the development of a variety of diseases, including: post-transplantation lymphoproliferative disorder (PTLD), acquired immune deficiency syndrome-related lymphoma (ARL), natural killer (NK)/T-cell lymphoma of T-cell origin, Hodgkin’s disease, Burkitt lymphoma (BL), Nasopharyngeal carcinoma (NPC) and EBV-associated gastric cancer (EBVaGC) of epithelial origin [1]. The significance of EBV in the transformation of human malignancies is still unknown, particularly in epithelial tumours [2]. For instance, most primary NPC cells were reported to gradually lose EBV during passages *in vitro*, raising uncertainty about the causal role of EBV in the oncogenesis of epithelial cancers [5]. The EBV-associated epithelial tumours, which account for 80% of all EBV-associated malignancies, have attracted researchers’ interest in recent years, particularly over the past two decades [6, 7]. These include NPCs and EBVaGCs, the most frequent EBV-associated epithelial tumours, with 78,000 and 84,000 new cases reported worldwide each year, respectively, among the 200,000 new cases of EBV-associated tumours [2, 8]. In these tumours, the clonal EBV genome and the expression of a subset of viral latent gene products are consistently found in virtually every cell. As a result, it has been hypothesised that EBV plays a critical role in the pathogenesis of these tumours [2, 9].

NPC is a distinct type of head and neck cancer, a squamous cell epithelia malignant tumour that originates from the lateral wall of the nasopharynx, notably in the fossa of Rosenmüller [7, 10, 11]. NPC has an unusual geographic and ethnic distribution, with particularly high incidence rates in South-East Asia and southern China (almost 80% of a total of 65,000 new cases) [7, 10, 11]. EBV is associated with over 98% of all NPC patients (100% of non-keratinizing NPC subtype) [2, 5]. Aside from that, approximately 10% of gastric carcinomas are associated with EBV (referred to as EBVaGC) and represent a disease that is relatively non-endemic, which is one of four subtypes of gastric cancer [12, 13]. The incidence of EBVaGC varies across the world, with a pooled estimate in Europe, Asia, North and South America of 9.2%, 8.3%, and 9.9%, respectively, whereas in Africa, a country like Zambia has an incidence of 23% [14]. Three histological subtypes of EBVaGC have been identified: conventional type adenocarcinoma (CA)-type, lymphoepithelioma-like carcinoma (LELC)-type, and carcinoma Crohn’s disease-like lymphoid reaction (CLR)-type [15], where ≥ 80% of EBVaGCs have morphology of LELC- type [10].

EBV exhibits type II latency in the majority, if not all, of NPC tumours, which is characterised by the expression of latent membrane protein 1/2 (LMP1 and LMP2), EBV-associated nuclear antigen-1 (EBNA1), EBV-encoded small RNA (EBER), and BamHI a rightward transcription (BART)-MicroRNAs (miRNAs), While EBVaGC is reported to exhibit latency I or II (mainly Latency I (EBNA-1, EBER and BART)) [14, 16, 17]. Several cellular processes and signalling pathways are believed to be disrupted as a result of the selective expression of EBV genes (type II latency), which contributes to the malignant transformation of epithelial cells. It has been demonstrated that EBV infection facilitates a distinct and alternate tumourigenesis process in epithelial malignancies by methylation pattern and a distinct mutation signature in the EBVaGC [18, 19]. Although the transition of premalignant epithelial cells into cancer cells by EBV is still debated, EBV has been demonstrated to exhibit oncogenic features such as increasing cell proliferation, angiogenesis, invasion, and chemotherapy resistance [7, 20].

It has been discovered that EBV encodes 44 mature miRNAs, which are transcribed into two regions around the BamHI fragment H rightward reading frame 1 (BHRF1) and BART, controlling both viral and cellular genes [21–23]. miRNAs are a type of small noncoding RNAs that range in length from 17 to 23 nucleotides and have a role in carcinogenesis and metastasis by forming imperfect complementary duplexes with their target mRNAs in the 3’-untranslated region (UTR), hence the expression of complementary mRNAs is transcriptionally reduced [24, 25]. BHRF1 encodes four mature miRNAs from three precursor miRNAs, whereas BART encodes forty mature miRNAs from 22 precursor miRNAs [21]. Because EBV miRNAs have been found in EBV-related tumours, it is believed that they play key roles in the pathobiology of the EBV life cycle and EBV-associated tumours. Several EBV-encoded microRNAs were found to aid the virus in evading immune detection by decreasing the expression of both host immune proteins and immunogenic viral antigens [25]. In addition, miRNAs encoded by EBV have a critical role in promoting viral replication and the development of EBV-associated malignancies by epigenetically regulating the expression of molecules that affect apoptosis, cell cycle progression, innate immunity, and migration [1]. It has been shown that the BART-encoded microRNAs (miR-BARTs) is abundantly expressed in EBV-associated epithelial malignancies and may play a role in the cell proliferation, promote malignant transformation, tumour metastasis [2, 26]. The use of such miRNAs as the diagnostic and prognostic biomarkers of EBV-associated tumours is presently being investigated [1]. Therefore, the discovery of EBV miRNAs may lead to the identification of novel therapeutic targets and the development of new therapeutic interventions for NPC and GC patients. This systematic review and meta-analysis assess the role of EBV miRNA in the prognosis of NPC and GC, as well as the response to treatment and survival outcome.

## Rationale

### The significance of the issue

MicroRNAs produced from the EBV gene BART are highly expressed in EBVaGC and NPC [27–29]. However, comparatively low levels of miRNAs-BART expression have been found in EBV-associated B-cell lymphoma and NK/T-cell lymphoma, indicating that BART miRNAs may play key roles in EBV-associated epithelial malignancies [30, 31]. Many studies have evaluated and reported the diagnostic, prognostic, and therapeutic significance of miRNAs-BART in EBVaGC and NPC through narrative reviews and research articles [22, 27, 32–36]. To the best of our knowledge, till now no quantitative and qualitative evaluation of miRNAs-BARTS on patient survival in EBVaGC and NPC through a systematic review and meta-analysis has been conducted. According to preclinical research, these miRNAs-BARTs play critical roles in the development, invasion, metastasis, and immune escape of NPC [28, 37–39]. A previous study discovered elevated plasma levels of miRNA-BARTs in NPC patients [40]. Previous research has shown that BARTs not only play important roles in the development of EBVaGC, but they may also act as prognostic biomarkers and possible therapeutic targets for EBVaGC [27]. Because there are no approved molecular biomarkers that are frequently accessible in clinical settings as promising prognosticators in EBVaGC and NPC, this study seeks to address this gap. This information might be beneficial in the management of EBV-associated epithelial cancers. As a result, we believe that a deeper understanding of EBV-miRNA expression in EBVaGC and NPC will provide a solid theoretical framework for EBVaGC and NPC treatment.

### How will this study address and help in this issue?

We conducted a systematic review and meta-analysis to address the significance of miRNAs-RARTs as potential prognosticators in NPC and EBVaGC, providing a framework for reporting the impact of miRNAs-BARTs expression on patient survival and identifying the pooled effect size of all NPC and EBVaGC prognostic studies. Obtaining a summary assessment of the association between increasing levels of miRNAs-RARTs expression and the risk of mortality by pooling the hazard ratio of all included studies will allow for a better understanding of the survival outcome of NPC and EBVaGC patients. This is the first study to conduct a qualitative and quantitative analysis of published articles on NPC and EBVaGC prognostic research and may be useful in investigating both the clinical and biological parts of the diseases at the molecular level. Given the unreliability of the current pathological prognostic biomarkers for NPC and EBVaGC, this research focuses on finding miRNAs-BARTs with prognostic and therapeutic value in NPC and EBVaGC. This research will also have clinical significance, perhaps assisting clinicians in treatment decision-making, to enhance preventative intervention and control, and providing better post-therapy care in NPC and EBVaGC, as well as enhancing clinical outcomes. These findings should also help to drive future clinical research and development in the field of NPC and EBVaGC prognosis.

## Methods

The study design was prospectively registered in PROSPERO, an online systematic review database (Record Number CRD42021269180).

### Search strategy

**Fig 1:**
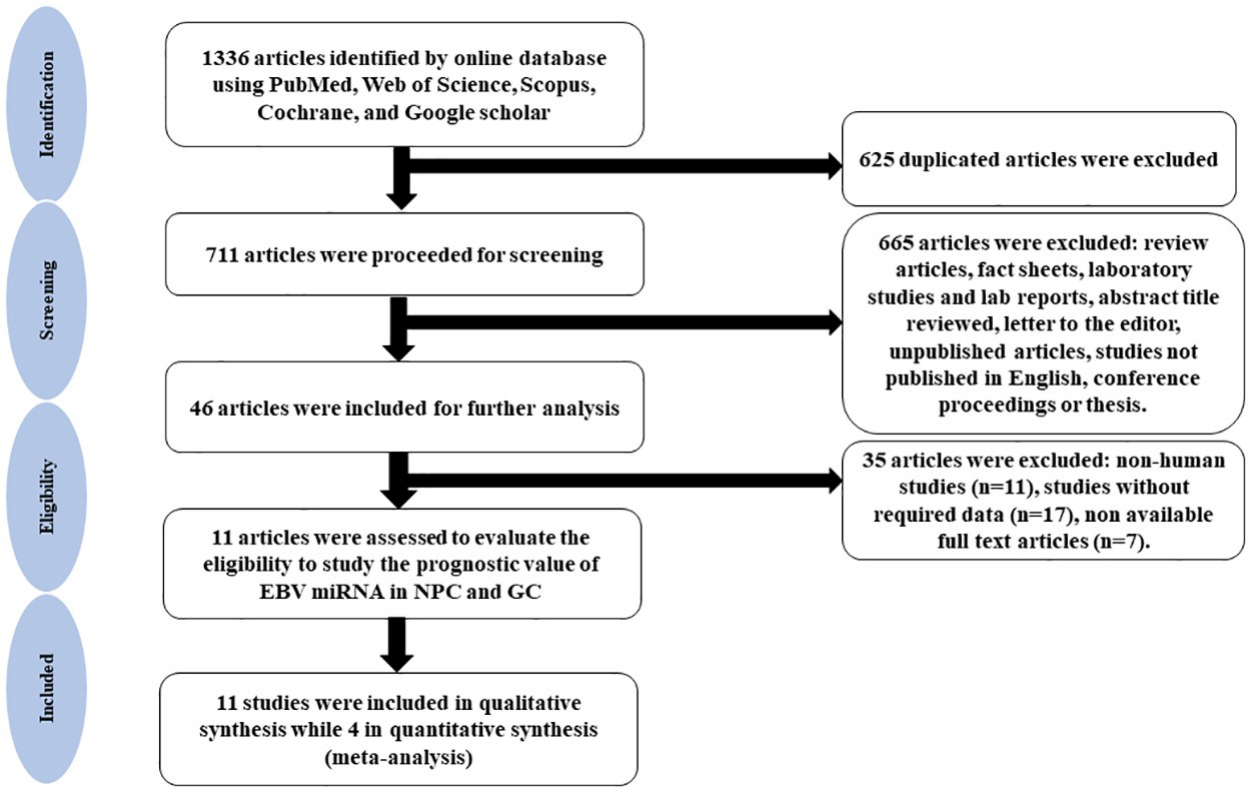
Flow chart of the study selection process.

To identify all published articles describing the significance of miRNAs-RARTs as potential prognosticators in NPC and EBVaGC, a rigorous literature search was done using PubMed, Web of Science, Scopus, Cochrane, and Google scholar. Databases were searched from the inception until July 24, 2021, with the limitation of solely selecting the publication in English language and the exclusion of any "grey literature (unpublished-informally published literature)". All the articles’ bibliographies obtained were examined for further relevant publications. The keywords utilized in this systematic review and meta-analysis were ((Epstein Barr virus) OR (Epstein-Barr virus) OR (EBV)) AND ((miRNA) OR (MicroRNA) OR (Mir-bart) OR (Bart) OR (EBV-Mir-Bart*) OR (MicroRNAs)). Two reviewers separately reviewed the titles and abstracts to find all eligible studies. To improve the robustness of the search, the reference lists of the selected articles were also examined. EndNote software version X8 (Thomson Reuters, California, USA) was used to import all records, and duplicate entries were cleared. Following consultation with a third reviewer, any disagreements between the two reviewers were settled by mutual conversation. The criteria defined in the Preferred Reporting Items for Systematic Review and Meta-Analysis (PRISMA) guidelines for systematic review and meta-analysis were applied to the retrieved articles. Fig 1 illustrates the selection and screening process of literature search using a flow chart.

### Selection criteria

The systematic review includes studies that met the following criteria.

### Inclusion criteria

1. Studies that discuss the prognosis of EBV miRNA in NPC and/or GC patients
2. Studies that discuss Hazard Ratio (HR) with 95% confidence interval (CI) for overall survival, disease-free survival, progression-free survival, distant metastasis-free survival and/or relapse-free survival as numerical data or KM curves.
3. Studies that interpret the overall survival, disease-free survival, progression-free survival, distant metastasis-free survival and/or relapse-free survival with univariate or multivariate analysis
4. Studies with the EBV miRNA expression profile and other clinicopathological parameters

### Exclusion criteria

1. Articles reporting the EBV miRNA expression profile on NPC and GC without patient survival data
2. Review articles, fact sheets, laboratory studies, letter to the editor and lab reports
3. Unpublished articles, studies not published in English and conference proceedings or thesis.

### Data extraction, management, and quality assessment

Two investigators (M.A.H.A. and A.A.I.) independently chose all the studies that were included based on titles and abstracts. If the publications were appropriate for the systematic review and meta-analysis, then the full text of the articles was retrieved. Furthermore, repeated screening of the excluded papers was done to ensure that no potentially relevant publications were missed. The information from published studies about NPC and GC patients’ clinical and treatment parameters were retrieved by two investigators (M.A.H.A and A.A.I). In addition, data on the type of sampling technique was gathered. For further evaluation of study quality and data synthesis, the collected data were recorded in a custom ‘Data Extraction Form’ created in Microsoft Excel.

The following information were extracted from the included articles: (1) information about the publication: authors’ names, area of publication, and year of publication; 2) patient’s characteristics: number of patients and clinical samples, type of cancer, disease stage, clinical sample type, histological type, lymph node metastasis/distant metastases, and years of follow-up; (3) NPC and GC patients’ survival data (HR and 95% CI), OS, DFS, PFS, DMFS, and/or RFS, and their 95% CI and p values.

A quality assessment tool developed by the National Heart, Lung and Blood Institute (NHLBI) for Systematic Reviews and Meta-analysis [41] was utilized to assess the methodological quality. This assessment tool was used to rate all the selected full-text articles as good, fair, or bad.

### Meta-analysis and assessment of heterogeneity

The meta-analysis was performed using Comprehensive Meta-Analysis (CMA) software to produce forest plots utilizing HR and associated 95% CI of OS, DFS, PFS, DMFS and/or RFS from the chosen studies. In meta-analysis, the mean effect estimate of HR is calculated more commonly than the sample size and statistical significance of included studies [42]. Because a low heterogeneity was anticipated, a fixed-effects model was used to estimate pooled HR value. Higgin’s I^2^ and Cochran’s Q-test were used to determine heterogeneity [43]. As a component of the statistical analysis carried out in the study, tau-squared statics was performed, which is the predicted variance between effects on test accuracy seen in different studies [44, 45]. If the P-value was less than 0.05, the results were considered statistically significant. The generated forest plots were evaluated and analysed to determine the effects of patient survival in NPC and GC. The NPC and GC groups were used in the subgroup analysis. In NPC and GC patients, HR >1 demonstrates upregulated expression of EBV miRNA which indicates a poor prognosis, while HR < 1 indicating a better prognosis.

### Publication bias

To report any publication bias, funnel plots and Egger’s bias indicator test were utilized. Funnels plots, which are scatter plots showing each study effect in proportion to their sample size, were created. A lack of skew or asymmetry indicates the absence of publication bias. When data from at least three studies are pooled, Egger’s regression intercept will be calculated. Egger’s test indicated the same bias by normalised effect estimate (estimate divided by standard error) vs precision (reciprocal of the standard error of the estimate) [46]. If there was a publishing bias, the Duval and Tweedie’s trim and fill method was applied to adjust the results. This method is a nonparametric method for estimating the number of missing studies in a meta-analysis and the impact these studies may have had on its outcome. Initially, this method used an iterative procedure in which it trimmed asymmetric studies from their right-hand side to find the unbiased effect, and then filled the plot with the right side’s trimmed studies, along with imputed counterparts to the left side [47].

## Results

### Study selection

Fig 1 is a schematic diagram of the study selection process. There were a total of 1336 publications found in the literature search by online database searches. After deleting duplicate records, 711 articles were screened. During screening, 665 items that were deemed ineligible based on language and article type were removed. The 46 remaining studies were then carefully evaluated by examining both the abstract as well as the full text of each study. Following that, 35 articles were further removed as they did not fulfil the inclusion criteria of our systematic review and meta-analysis.

Finally, 11 studies were analysed to evaluate the potential association between EBV miRNA expression and NPC and GC prognosis (Table 1). Following a comprehensive examination of the 11 full-text papers against the stated inclusion criteria, seven articles did not provide HR values and/or 95% CI, thus, four studies [13, 28, 35, 48] discussing the prognostic importance of miRNAs in NPC and GC patients were included in the meta-analysis. In addition to reporting the HR and 95% CI values, the four articles also provided other necessary data including survival data in the form of Kaplan Meier curves (Table 1).

**Table 1:** Characteristics of studies included in the systematic review and meta-analysis.

No of Study, [reference]	Population	Gender	Sample Size	Sample Source	Platform	Follow-Up Period	EBV miRNA	Histopathological Type	Lymph Node Metastasis/ Distant Metastasis	Cancer Type/ Subtypes	Endpoint	HR Value	EBV miRNA Dysregulation	Disease	Significant	Not Significant
Chan et al.,2015 [49]	China	F = 25/ M^a^ = 77	102	Tissue	qPCR	52–96 months	miR-BART7	N/A	N/A	T1-T4	Probability of local tumour recurrence and PFS	Cox proportional- hazards model for multivariate analysis and KM curve	Upregulated	NPC	The miR-BART7 was significantly associated with local tumour recurrence with clear restriction margins (≥ 5); HR = 6.834; *P* = 0.01.	The miR-BART7 was not significantly associated with PFS in patients with clear restriction margins (≥ 5)
Yan at al., 2015 [22]	China	F = 19/M^a^ = 87	106	Tissue biopsy	ISH, IHC, northern blots and qPCR	120 months	miR-BART10-3p	WHO type II	N0-N1/M0-M1	T1-T4	OS, RFS, DMFS and DFS	KM curve	Upregulation	NPC	The miR-BART10-3p was significantly associated with OS, RFS, DM and DFS; RFS or DMFS (*P* < 0.05), DFS (*P* = 0.030) and OS (*P* = 0.010). The HR values were not reported for the survival endpoint.	-
Kang et al., 2017 [13]	South Korea	F = 12/ M^a^ = 47	59	Tissue	qPCR	2.8–48.0 months	miR-BART1-5p, miR-BART4-5p, and miR-BART20-5p	1-GCLC 2- non- GCLC	N0-N3 M^b^: N/A	T1-T4	RFS and OS	KM curve	Upregulated	EBVaGC	The miR-BART20-5p was significantly associated with worse RFS (*P* = 0.034, HR = 6.951, 95% CI of HR = 1.158–41.737)	The miR-BART1-5p, miR-BART4-5p were not Significant
Liu et al., 2019 [50]	China	F = 55/ M^a^ = 108	163	Tissue	RT-qPCR and ISH	N/A	miR-BART22	N/A	N0-N3/M0-M1	T1-T4	OS	KM curve	Upregulated	NPC	The miR-BART22 was significantly associated with OS (*P* = 0.003). The HR value was not reported for the OS endpoint	-
Dong et al., 2020 [27]	China	F = 5/ M^a^ = 66	71	Tissue	qRT-PCR and western blotting	N/A	miR-BART10-3p and miR-BART22	Differentiation: 1-Well/Moderate 2-Poor	N: Negative/ Positive M^b^: Negative/ Positive	T1-T4	OS	KM curve	Upregulated	EBVaGC	The miR-BART10-3p and miR-BART22 were significantly associated with OS (*P* = 0.003 and *P* = 0.025, respectively). The HR value was not reported for the OS endpoint	-
Jiang et al., 2020 [48]	China	F = 32/ M^a^ = 118	150	Serum	qRT-PCR	N/A	miR-BART2-5p	UNPC	N0-N3/ M0-M1	T1-T4	PFS	KM curve and Multivariate survival analysis	Upregulated	NPC	The BART2-5p 6 was significantly associated with PFS (HR = 2.184, 95%CI of HR = 1.119–4.263, *P* = 0.022).	-
Lu et al., 2020 [28]	China	F = 177/ M^a^ = 339	465	Plasma	qRT-PCR	55 (2–83) months	miR-BART7-3p and miR-BART13-3p	1- Keratinizing squamous cell 2- Nonkeratinizing, differentiated 3- Nonkeratinizing, undifferentiated	N0-N3 M^b^: N/A	T1-T4	DMFS	KM curve	Upregulated	NPC	The BART7-3p was significantly associated with DMFS (HR = 2.14, 95%CI of HR = 1.04–4.42, *P* = <0.001)	The BART13-3p was not significantly associated with DMFS.
Mo et al., 2018 [51]	China	F = 25/ M^a^ = 64	89	Tissue	qPCR	4 to 82 months	miR-BART8-3p	N/A	N: N/A M^b^: yes/ no	N/A	OS	KM curve	Upregulated	NPC	The EBV-miR-BART8-3p was significantly associated with worse OS (*P* = 0.007). The HR value was not reported for the OS endpoint.	-
Wu et al., 2020 [36]	China	F = 38/ M^a^ = 121	195	Plasma	qRT-PCR	N/A	miR-BART19-3p	1-PDSCC 2-Non-keratinizing carcinoma 3-NA	N/A	N/A	OS	KM curve and Cox’s proportional hazards model analysis	Upregulated	NPC	-	The miR-BART19-3p was not significantly associated with OS.
Wu et al.,2020 [52]	China	N/A	27	Tissue	ISH, IHC and qPCR	N/A	miR-BART12	N/A	N/A	N/A	OS	KM curve	Upregulated	NPC	-	The miR-BART12was not significantly associated with OS
Tang et al.,2021 [35]	China	F = 23/M^a^ = 73	96	Serum	qRT-PCR	3–89 months	miR-BART6-5p	N/A	N0-N3 M^b^: N/A	T1-T4	OS	Multivariate Cox regression analysis and KM curve	Upregulated	NPC	The miR-BART6-5p was significantly associated with OS (HR = 3.887, 95%CI of HR = 1.243–12.15, *P* = 0.020)	-

F, female; M^a^, male; ISH, in situ hybridization; IHC, immunohistochemistry; qPCR, real-time PCR; T, primary tumour; qRT-PCR, quantitative reverse transcription PCR; N, regional lymph nodes; M^b^, distant metastasis; PFS, progression-free survival; OS, overall survival; DFS, disease-free survival; DMFS, distant metastasis-free survival; RFS, relapse-free survival; UNPC, undifferentiated non-keratinizing carcinoma of nasopharyngeal type; GCLC, gastric carcinoma with lymphoid stroma; PDSCC, poorly differentiated squamous cell carcinoma; NPC, nasopharyngeal carcinoma; EBVaGC, Epstein-Barr virus associated gastric cancer; KM curve, Kaplan Meier curve.

### Characteristics of the included studies

Table 1 summarizes the main characteristics of the included studies. The cancers considered in this analysis were NPC and GC. Across all studies, the 11 studies for qualitative synthesis included 1,393 NPC and 130 GC patients, while the 4 studies for quantitative synthesis (meta-analysis) included 711 and 59 of NPC and GC patients, respectively. The cohort sizes of the 11 studies ranged from 27 to 465 participants, while the cohort size for the 4 studies that were included in meta-analysis ranged from 59 to 465 participants. All included studies were conducted in China, except one study which was conducted in South Korea. Real-time PCR (qPCR) and quantitative reverse transcription PCR (qRT-PCR) were used to detect EBV miRNA expression.

Among four included studies, EBV miRNAs were extracted from tissue (one research), plasma (one study), and serum samples (two studies). There was one study that did not give data on the follow-up period, while the average follow-up periods from other studies ranged from two to 89 months. Across all included studies, the seven miRNAs were discovered to be upregulated. The HR values and 95% CI in the current analysis were obtained from multivariate analysis (in two studies) and Kaplan-Meier analysis (in all included studies) which were provided by the included studies.

### Comprehensive meta-analysis and survival outcome

**Fig 2:**
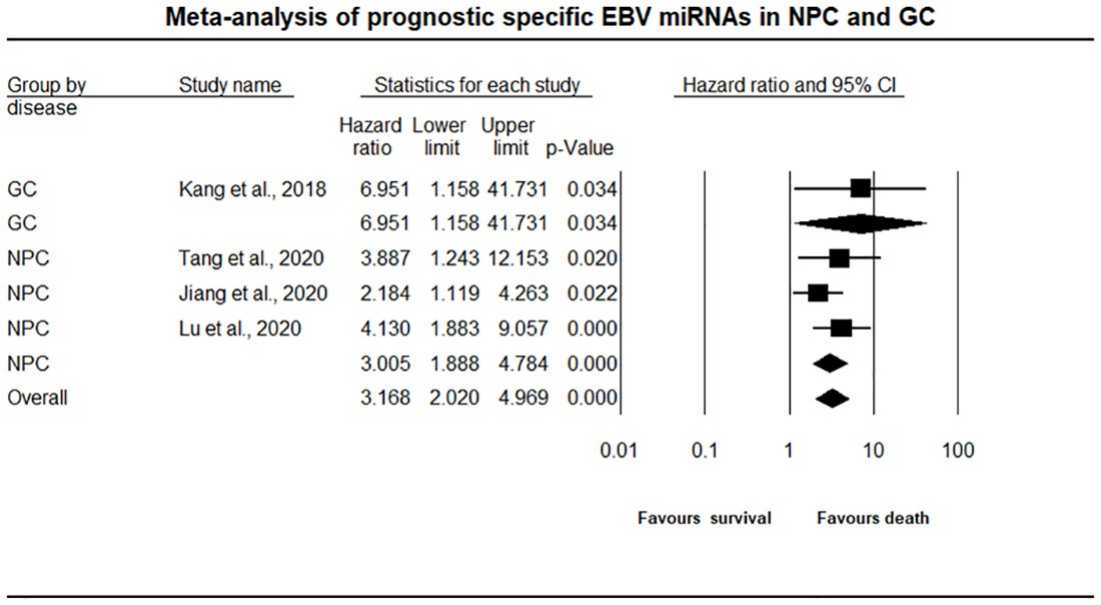
The forest plot of EBV miRNA survival outcomes in NPC and GC patients. CMA software (version 3.3.070, USA) was used to construct and assess the pooled hazard ratios of HR values for NPC and GC prognostic data. The black square with line indicates the estimated pooled effect of survival for NPC and GC patients randomly allocated to EBV miRNA evaluation. The effect size of EBV miRNA in the included studies was represented by a black diamond, with a 95% confidence interval. A HR of one indicates that there is no difference in the risk of NPC or GC patients’ survival. A HR more than one implies an increased risk of patient survival, whereas a HR less than one indicates a lower risk of patient survival.

The meta-analysis results were based on the survival data of 711 NPC and 59 GC patients, assessing a total of seven EBV miRNAs as possible prognostic biomarkers which were reported across the four individual EBV miRNA studies (Fig 2). All the seven miRNAs were found to be upregulated. Among NPC and GC Patients, the OS was reported in all four studies, and three studies also examined the PFS or DMFS or RFS. In the fixed effects model, the overall pooled effect estimate of HR for EBV miRNA expressions (in both NPC and GC patients) was 3.168 with a 95% confidence interval of 2.020–4.969, where the estimated pooled HR value for GC and NPC subgroups was 6.951(95% CI:1.158–41.731) and 3.005 (95% CI: 1.888–4.784), respectively, as shown in Fig 2 and Table 2. However, the estimated pooled HR value of GC subgroup is contributed by one study. In this meta-analysis, the overall pooled HR estimate indicated that upregulated EBV miRNA expression increased the risk of mortality and lead to poor survival in NPC and GC patients by three times. Table 2 demonstrates the heterogeneity, publication bias and hypothesis testing.

**Table 2:** The heterogeneity, publication bias and hypothesis testing of the included studies in the meta-analysis.

Publication bias																
					Fixed			Mixed			Hypothesis Test					
Subgroup	Heterogeneity				HR	95% CI		HR	95% CI		Fixed Effects Model			Mixed Effects Model		
	Q-value	P-value	df (Q)	I ^2^		Lower limit	Higher limit		Lower limit	Higher limit	Z-value	P-value	Number of studies	Z-value	P-value	Number of studies
**GC**	**0.000**	**1.000**	**0**	**0.000**	**6.951**	**1.158**	**41.731**	**6.951**	**1.158**	**41.731**	**2.120**	**0.043**	**1**	**2.120**	**0.043**	**1**
**NPC**	**1.700**	**0.4247**	**2**	**0.000**	**3.005**	**1.888**	**4.784**	**3.005**	**1.888**	**4.784**	**4.639**	**< 0.001**	**3**	**4.639**	**< 0.001**	**3**
**Total within**	**1.700**	**0.427**	**2**	**-**	**-**	**-**	**-**	**-**	**-**	**-**	**-**	**-**	**-**	**-**	**-**	**-**
**Total between**	**0.788**	**0.375**	**1**	**-**	**-**	**-**	**-**	**-**	**-**	**-**	**-**	**-**	**-**	**-**	**-**	**-**
**Overall**	**2.488**	**0.477**	**3**	**0.000**	**3.168**	**2.020**	**4.969**	**3.168**	**2.020**	**4.969**	**5.023**	**< 0.001**	**4**	**5.023**	**< 0.001**	**4**

#### Does EBV miRNA expression affect the survival of NPC and GC patients?

The Z values for the test of null hypothesis (mean hazard ratio is 1.0) were 2.120 and 4.639, with corresponding P-values of 0.043 and < 0.001 in the GC and NPC subgroups, respectively, indicating that an increased risk of death is associated with an upregulated EBV miRNA expression level in both subgroup (Table 2). From our findings, we can infer that the risk of death will increase with the upregulated EBV miRNA expression level. The upregulation of miR-BART6-5p, miR-BART2-5p, miR-BART7-3p, and miR-BART13-3p, miR-BART1-5p, miR-BART4-5p, and miR-BART20-5p, was correlated with poor patient survival, presumably suggesting a poor prognosis. Because of the low level of heterogeneity in the data, a fixed-effects model was employed to determine and analyse the pooled HR value. In patients with NPC and GC, a high level of EBV miRNA expression was related with a poor OS, PFS, RFS and DMFS.

#### What is the extent to which the estimated effect size of NPC and GC patients varies among the studies included in this analysis?

The Q-statistic is used to test the null hypothesis, which states that all studies in the analysis have the same effect size. All studies with similar effect sizes would have an expected Q value equal to the degrees of freedom (df) (the number of studies minus 1), where the Q-value was 2.488, and the p-value was 0.477. Because the observed variation falls within the range that may be related to sampling error, we are unable to reject the null hypothesis that the actual effect size was the same of all studies.

In addition, we can proceed to assess the extent of the variation that has occurred. Using the I^2^ statistic, we can determine what proportion of the observed variance is due to differences in true effect sizes as opposed to sampling error. I^2^ was 0% in this case. The variance of true effect sizes is represented by T^2^ (in log units). T^2^ was 0.000 in this case. The standard deviation of actual effects is represented by the letter "T = tau" (in log units). T was 0.000 in this case.

#### Is there a difference in the extent of the effect depending on subgroup (NPC and GC)?

It’s probable that the mean hazard ratio varied by subgroup, even though the mean effect size of all studies was moderate (with a hazard ratio of 3.168). With the use of the subgroup analysis, we were able to compare the effect size in studies that determined the EBV miRNA expression in NPC and GC patients, where the mean hazard ratios were 3.005 and 6.951, respectively. The Q value for the differences was 0.788, and the P value was 0.375. Therefore, there was no indication that the hazard ratios differed as a result of NPC or GC patients’ survival.

### Publication bias and sensitivity analysis

#### Funnel plot

The funnel plot of overall studies spanning the survival outcomes is depicted in S1 Fig, which is asymmetric. In this case, the funnel plot’s asymmetry indicates the presence of publication bias. It is possible that the asymmetry is due to small-study impacts (such as sampling error).

#### Egger’s bias indicator test

In this analysis, the intercept (B0) was determined to be 1.85625, 95% CI (-2.91581–6.62832), with t-value = 1.67366 and df = 2.00000. The suggested 1-tailed p-value was 0.11809 and 2-tailed p-value was 0.23617.

#### Duval and Tweedie’s trim and fill

With the use of these parameters, the method identified one missing study. The funnel plot for the trimmed and imputed study was created with using CMA software (S2 Fig). According to the fixed effect model, the point estimate and 95% CI for the included studies were 3.16825 (2.02015–4.96884). Trim and Fill method was used to obtain an adjusted point estimate of 3.00513 (1.94228–4.64961).

We focused on the effectiveness of employing EBV miRNA as prognostic biomarkers in patients suffering from NPC and GC based on the findings of 11 previously published studies. A necessity for accurate prognosis of NPC and GC is standardisation of particular EBV miRNA. Hence, large-scale prospective observational clinical research is required to measure the inter-observer variability and repeatability.

### Quality assessment of the selected studies

Table 3 summarizes the criteria and methodology rating. The quality assessment of each study was reported in S1 Table, where most of the studies (10/11) achieved good quality scores, suggesting that the included studies were of good methodological quality, while one received fair score. Table 3 demonstrated the quality scores based on the criteria of quality assessment developed by the National Heart, Lung and Blood Institute (NHLBI) for Systematic Reviews and Meta-analysis [41]. The scores ranged from good to fair. The cardinal score (Good-4 studies, Fair-7 studies) was assigned based on the results of the HR and 95% CI values, which were critical for this investigation.

**Table 3:** Selected studies’ quality assessment based on criteria of quality assessment.

No	Criteria	Good (67–100%)	Fair (33–66%)	Bad (0–33%)
**1**	Research question or the objective of this paper clearly stated	11 studies	-	-
**2**	Study population clearly specified and defined	11 studies	-	-
**3**	Participation rate of eligible persons at least 50%	11 studies	-	-
**4**	Eligibility criteria	11 studies	-	-
**5**	Sample size justification, power description and effect estimates	-	-	11 studies
**6**	EBV miRNA exposure assessed before outcome measurement	11 studies	-	-
**7**	Timeframe sufficient for the patients (OS, PFS, RFS or DMFS)	10 studies	1 study	-
**8**	Different levels of the exposure of interest as related to the outcome (low or high expression of EBV miRNA)	9 studies	2 studies	-
**9**	Exposure measures clearly valid, defined, and implanted of all study participants (tool or method used to measure exposure)	11 studies	-	-
**10**	Repeated exposure assessment (more than once over time)	11 studies	-	-
**11**	Outcome measures (HR, and CI)	4 studies	7 studies	
**12**	Blinding of outcome assessors	NA	NA	NA
**13**	Follow-up rate	10 studies	1 study	-
**14**	Statistical analysis	11 studies	-	-
	**Total selected studies**	4 studies	7 studies	-

## Discussion

Several research and narrative reviews have reported the significant role of EBV miRNA as prognostic biomarker of NPC and GC patients and in predicting the survival outcome [13, 22, 25, 52–55]. The goal of this systematic review and meta-analysis is to determine the statistical association between EBV miRNA expression levels and the survival outcome of NPC and GC patients. The EBV miRNA expression variation has been evaluated in relation to gender and other clinical and pathological features in the studies selected in our systematic review [13, 22, 27, 28, 35, 36, 48–52].

Asia has the largest number of NPC new cases and overall mortality rates for males and females [56]. In both NPC-endemic and non-endemic regions, age-standardised rate (ASR) are consistently 2–3 times higher in males than in females [57]. Males were more prominent in the development of NPC compared to females with a 3:1 ratio [56]. Differences in NPC rates between men and women may be due to different lifestyle patterns (e.g., intake of tobacco) or biological differences [58, 59]. To date, only a few studies have explicitly focused on the prognostic effect of gender and age on NPC. It was also noted that male patients were more likely to have distant metastasis, have a shorter time before therapeutic failure, had lower OS rates and PFS rates relative to female patient [60]. Male gender has been found to be strongly associated with EBVaGC in most meta-analyses, where the male to female ratios were reported as 2:1 or 3:1 [61]. The EBVaGC were shown to be significantly related with gender, where male appear to be a risk factor for poor prognosis [62]. Among the included studies in our systematic review, three studies investigated the relationship between a patient’s gender and prognosis [28, 35, 51]. However, the effect of gender on patient survival was not statistically significant in these included studies.

A bimodal pattern of age distributions has been observed in low-incidence populations, with an initial increase in early adulthood accompanied by a second peak later in life [63]. On the other hand, the highest occurrence in high incidence populations is between 45 and 59 years of age, and it appears that the early exposure to carcinogens, such as childhood environmental factors and genetic predisposition, is important in the aetiology of WHO NPC type II / III [63, 64]. Previous studies reported that age was a major prognostic factor that influenced NPC patients’ survival [60, 65–67], where that younger patients had slightly greater survival and better prognosis compared to older patients [67]. On the other hand, EBVaGC was more prevalent among younger patients, in accordance with previous findings [68, 69]. The Ethnicity and lifestyle variables impact the distribution of EBVaGC [15]. It was discovered that younger patients had a better prognosis in terms of cancer-related survival and DFS, and lower tumor-node-metastasis system-stage [70, 71]. Our systematic analysis includes four studies that investigated the relationship between a patient’s age and prognosis in NPC (three studies) and GC (one study) [13, 28, 35, 51]. The effect of age on patient survival was not statistically significant in these included studies. Furthermore, smoking [35, 49, 50], family history [35, 50], drinking and treatment [35] may be considered risk factors for NPC.

Up to 40 mature EBV miRNAs have been found until now [72]. These viral miRNAs are probably involved in tumour development and progression. Furthermore, various studies have explored the function of these miRNAs as biomarkers for early diagnosis, therapy response and prognosis [49].The expression of EBV BART miRNAs may be found in all forms of latency, but they are especially abundant during latency I and II in epithelial hosts. A significant proportion of the total EBV miRNAs in infected NPC or GC epithelial cells and tissues is comprised of BART miRNAs, indicating that EBV BART miRNAs may play a role in the progression of epithelial malignancies [73–75]. Even though research on EBV miRNAs is progressing, there are still significant limitations in the studies. Due to the lack of a suitable animal model for EBV-associated tumours, substantially all research on the function of BART miRNA relies on EBV-infected cancer cell lines, which is a significant limitation [76].

A significant number of BART miRNAs, such as miR-BART10-3p, miR-BART1-5p, miR-BART7-3p, and miR-BART6-5p, were found to be significantly increased in NPC, indicating these miRNAs may function as an oncogene and prognostic biomarker and may have a role in the occurrence and progression of NPC [22, 77–79]. It has been shown that EBV-miR-BART1 has a direct effect on the expression of NPC mechanism-associated genes such as phosphoserine aminotransferase 1 (pSAT1), phosphoglycerate dehydrogenase (PHGDH) and phosphatase and tensin homolog (PTEN) [77, 80]. This miRNA downregulates PTEN expression, resulting in an increase in NPC invasion, migration and metastasis by enhancing epithelial-mesenchymal transition (EMT) signalling pathways [81]. NPC patients with high EBV-miR-BART10-3p expression were shown to have a poor prognosis [22], where EBV-miR-BART10-3p might enhance NPC cell migration and invasion, through β-Transducin Repeat Containing E3 Ubiquitin Protein Ligase (BTRC) inhibition [22, 82, 83]. miR-BART6-5p has been identified as a prognostic factor in NPC cells, whereby this miRNA contributes to the migration, proliferation and invasion of NPC cells, which is followed by the suppression of apoptosis as well as the lowering of radio-sensitivity, ultimately leading to a poor prognosis and OS [22]. BART7- 3p expression in the resection margins of recurrent NPCs indicates a greater risk of local tumour relapse in NPC [49], and can also use as potential therapeutic targets for NPC [84]. Cancer stemness, chemoresistance and metastasis are all promoted by EBV-miR-BART22 as an oncogene biomarker [50, 85, 86]. In NPC clinical samples, miR-BART22 was found with high expression levels, which correlated with clinical progression, a poor prognosis in NPC patients and poor predictor of OS and DMFS [28, 50].

Among NPC patients, significant level of expression of EBV-miR-BART12 were reported and patients with low expression levels of EBV-miR-BART12 had a considerably lower OS than patients with high EBV-miR-BART12 expression; hence this miRNA was correlated with poor prognosis [52]. According to previous findings, BART12 enhances nasopharyngeal carcinoma cells transformation into a mesenchymal-like form, thereby promoting the metastasis and invasion of NPC. It also increases the mitosis of cancer cells, thereby improving the capacity of cancer cells to transfer and invade [52, 87, 88]. In addition, BART12 was shown to be substantially expressed in both EBVaGC tissue and cell lines by Kim and colleagues [89]. According to previous research, the BART2-5p was shown to be positive in most NPC patients but not in healthy controls [90, 91]. Even though BART2-5p has been shown to promote immune escape from natural killer cells (NK cells) [92] and to prevent EBV transition from latent to lytic viral replication [93], its biological involvement in the development of NPC has remained unclear [48]. It was discovered that patients with a higher expression of BART2-5p in their blood were more likely to develop recurrence, metastasis [94] and had a shorter PFS, where this miRNA acts as an independent negative prognostic factor in NPC patients [48]. Furthermore, in patients with nasal NK/T-cell lymphoma, a high BART2-5p level was shown to be substantially associated with a poor overall survival rate and disease progression [95].

BART13 has been discovered in high concentrations in the plasma of NPC patients, while it has been found in low concentrations in the plasma of non-NPC patient populations or healthy controls. Furthermore, a decrease in BART13 levels in plasma following radiotherapy suggests that they may be good indicators of treatment effectiveness [40]. Even though miR-BART13-3p had a lower prognostic value than miR-BART7-3p in NPC patients, two studies conducted *in vitro* and *in vivo* have revealed that miR-BART13-3p can enhance the NPC cells metastasis by decreasing the expression of Abl interactor 2 (ABI2) and NF-κB inhibitor interacting Ras-like 2 (NKIRAS2) and the pro-apoptotic protein caprin family member 2 (CAPRIN2) [40, 96, 97]. However, it appears that biological behaviour may not be the underlying reason miR-BART13-3p is not a significant independent prognostic factor in NPC disease [28]. Patients with nasal NK/T-cell lymphoma have been shown to have increased levels of BART13-3p in their serum when compared to healthy controls, thus, it can be used as potential prognostic and diagnostic biomarkers in nasal NK/T-cell lymphoma patients [97]. MiR-BART19-3p expression levels in plasma were shown to be considerably higher in NPC patients [36, 98]. Even though, this miRNA serves as a diagnostic biomarker for NPC, no significant correlation between this miRNA expression level and prognosis of NPC has been established [36]. MiR-BART19-3p, on the other hand, was associated with reduced apoptosis and increased proliferation in NPC cells via modulating the Wnt pathway by targeting inhibitory genes of Wnt pathway such as adenomatous polyposis coli (APC), Wnt inhibitory factor 1 (WIF1) and Nemo‐like kinase (NLK) [36, 98–100].

EBV-miR-BART1, EBV-miR-BART7, and EBV-miR-BART8-3p were found to be significantly expressed in NPC and were linked to pathological and advanced clinical stages of the disease [52, 77, 101]. Changes in these miRNA expressions promote an increase in migration and invasion of NPC cells *in vitro*, resulting in tumour metastasis [51, 77, 101]; hence, these BART miRNAs will lead to distant metastasis, poor prognosis, and poor OS [51]. Several studies discovered that plasma EBV miR-BART7 levels were considerably higher in patients with NPC, particularly those with advanced stages [40, 101, 102]. Their levels were dramatically lowered following radiotherapy treatment, making them potential biomarkers for diagnostic and treatment effectiveness prediction [40]. Among NPC patients with clear histologic margins, positive EBV miRNA BART7 status were associated with higher probability of local tumour recurrence following salvage nasopharyngectomy [49].

BART10-3p and BART22 were found to be overexpressed in EBVaGCs [14, 73] and were linked to lymph node metastasis and a worse 5-year OS in GC patients, and can be used as potential therapeutic targets and prognostic biomarker for EBVaGC [27]. Further investigation revealed that BART10-3p and BART22 contributed to the metastasis and invasion of EBVaGC cells [27]. Upregulation of BART20-5p in tumour tissues has been linked to worse 3-year RFS in EBVaGC, but no association was established with OS or other clinicopathological characteristics [13]. Researchers found that patients with lower miR-BART20-5p expression had a better RFS than those who had higher levels of miR-BART20-5p [13, 103]. The current findings, which reveal an inverse connection between survival and miR-BART20-5p expression levels, imply that miR-BART20-5p may operate as a potential therapeutic target in EBVaGC [13]. Other investigations have found that EBV miRNAs have clinical significance in EBV-associated malignancies. A high expression of BART8 and BART20-5p has been identified simultaneously among a group of invasive Nasal NK-cell lymphomas [104].

Shinozaki-Ushiku et al. found that the expression of miR-BART4-5p, which suppresses the proapoptotic protein Bid (pro-apoptotic Bcl-2 protein), reduced apoptosis in clinical samples from EBVaGC patients. These findings may indicate that BART miRNAs may be involved in activating numerous important cancer-related proteins [14]. Nonetheless, despite the elevated expression of miR-BART1-5p and miR-BART4-5p, the prognostic importance of these miRNAs was not well defined. The oncogenic significance of BART1-5p was examined utilizing NPC and lymphoblastoid cell lines in a recent miRNA profiling investigation. Consequently, miRNA-BART1-5p was discovered to inhibit the target gene in EBV as well as the host cellular protein [103, 105]. Furthermore, suppressing BART4-5p expression has been linked to the partial recovery of the apoptotic activator molecule in EBVaGC. It is possible that BART miRNA alterations are implicated in various mechanisms in various tumour types [106].

## Strength

As a result of this study’s extensive systematic database searches and comprehensive analytical approach, the most up-to-date information on EBV miRNA prognosticators for NPC and GC, based on newly published studies from around the world, was reported. Because the studies included in this analysis were selected from all presently available NPC and GC studies that investigated EBV miRNA expression and prognosis, the combined effect size of all selected studies might be used to any future NPC and GC survival studies that will be conducted. A valid methodology quality assessment was performed on the included NPC and GC studies, and most of the studies were determined to be with a good quality. The goal of this systematic review and meta-analysis is to investigate and determine the usefulness of EBV miRNA as prognostic biomarkers in both NPC and GC. This is the first study to conduct a systematic review and meta-analysis on the quantitative analysis of EBV miRNAs in epithelial cancers (NPC and GC). Furthermore, all research in this analysis was conducted in accordance with the PRISMA guidelines.

## Limitation and future direction

Because the studies included in the meta-analysis were only conducted in two countries (China and South Korea), the paper’s worldwide clinical applicability may be restricted. By utilizing authentic and widely used data bases in this meta-analysis, the literature searches discovered studies that focused primarily on Asian populations and did not include a significant number of other populations, such as Caucasians. As a result, our findings of EBV miRNA as a potential prognostic biomarker in NPC and GC may not be applicable to other populations.

The minimal number of studies included in this meta-analysis was a significant limitation of this study, which should be noted. A small number of studies frequently restricts the analysis’s capability to be used in a broad range of clinical settings. Because of the small sample size of 711 NPC and 59 GC patients and particularly due to only one study contributing to pooled HR value for GC subgroup, the statistical significance of EBV miRNA expression levels as prognostic biomarker may be reduced. Consequently, to determine the role of EBV miRNAs in predicting survival outcomes of NPC and GC patients, large-scale, multi-center prospective clinical studies should be conducted in the future, particularly in the context of multimodal treatment and the high risk of recurrence after therapy, even in early-stage diagnosis, which also may be useful in future efforts to conduct an updated systematic review and meta-analysis. The current state of knowledge on prognostic biomarkers that can reliably provide predictive survival data for NPC and GC is insufficient. Hence, more research is needed to determine the carcinogenesis roles of EBV BART miRNAs in GC and NPC, as well as their potential as therapeutic targets and prognostic biomarkers. Therefore, a recent systematic review and meta-analysis based on the extensive clinical data sets may have an impact on determining the aggressiveness of therapy, the sequence of treatments and, ultimately having a favourable impact on the survival of NPC and GC patients.

The asymmetrical funnel plot results suggested that there was a considerable publication bias in this meta-analysis of four studies. While this bias may be apparent, we adjusted for it using the Trim and Fill procedure to ensure that our results are more reliable. A slight reduction in the HR ratio was however observed.

We were unable to include some of the investigated studies because the HR and CI values were not provided; thus, these studies were not included in this analysis to investigate the impact of EBV miRNAs on survival outcomes. Due to the lack of available HR and CI data in many of the included studies, the effects of EBV miRNAs based on patient-related variables, clinicopathological-related variables, treatment-related variables, and other clinical outcome variables were not possible. In addition, we were unable to do any additional subgroup analysis due to the reported parameters and the small number of available studies.

## Conclusion

This systematic review and meta-analysis provide more support for hypothesis that EBV miRNAs play a critical role in the prognosis of NPC and GC patients. Our study identified seven EBV miRNAs that have the potential to serve as prognostic biomarkers in NPC and GC. According to our findings, higher EBV miRNA expression is related with a poor survival outcome in patients with NPC and GC. However, more extensive studies and analyses are required before the EBV miRNAs may be used therapeutically in clinical settings against NPC and GC. Furthermore, it recommends that researchers must focus on large-scale cohort studies to validate the EBV miRNAs as prognostic biomarker, rather than small-scale studies. Comprehensive clinical research with large sample sizes is required to further understand the applicability of EBV miRNAs in the clinical setting, as well as to validate and assess the use of EBV miRNAs as prognostic biomarkers and therapeutic target, and to establish their influence on NPC and GC patient survival outcomes.

## Supporting information

S1 Checklist PRISMA checklist items. (PDF)

S1 Table The overall quality assessment of each study. (PDF)

S1 Fig Funnel plot of standard error by log hazard ratio associating NPC and GC patients’ survival with EBV microRNA expression. The funnel plot depicts the precision of the study size and standard error on the vertical axis as a function of the effect size on the horizontal axis. The individual study is represented by a dot, and most of this area comprises regions of high significance, indicating that publication bias would be reflected by asymmetrical distribution of dots. The smaller studies were represented at the bottom of the funnel plot at the bottom of the, where these studies are more likely to be published if they have larger-than-average effects, making them more likely to achieve the statistical significance criterion.
(PDF)

S2 Fig Funnel plot containing observed and adjusted studies. The Smaller studies are clustered at the bottom of the graph, and they will be distributed throughout a wide range of values because of the greater sampling variation in effect size estimates in the smaller studies. The funnel plot displays the precision and standard error of the study size on the vertical axis as a measure of the effect size on the horizontal axis. Individual studies are displayed by dots, and most of this area comprises regions of high significance, showing that publication bias is shown as asymmetrical graph. If smaller studies (those that appear at the bottom of the funnel plot) exhibit larger-than-average effects, they are more likely to be published, and they are more likely to achieve the statistical significance criterion, as would be expected.
(PDF)
