# Supplementary material for: Prognostic Epstein-Barr Virus (EBV) miRNA biomarkers for survival outcome in EBV-associated epithelial malignancies: Systematic review and meta-analysis
Source: PLoS One. 2022 Apr 18;17(4):e0266893. doi: 10.1371/journal.pone.0266893 (PMC9015129; doi:10.1371/journal.pone.0266893)
Supplement: S1 Table — (PDF) [file pone.0266893.s002.pdf]

S1Table: The overall quality assessment of each study.

Study	Research question or the objective of this paper clearly stated	Study population clearly specified and defined	Participation rate of eligible persons at least 50%	Eligibility criteria	Sample size justification, power description and effect estimates	EBV miRNA exposure assessed before outcome measurement	Timeframe sufficient for the patients (OS, PFS, RFS or DMFS)	Different levels of the exposure of interest as related to the outcome (low or high expression of EBV miRNA)	Exposure measures clearly valid, defined, and implanted across all study participants (tool or method used to measure exposure)	Repeated exposure assessment (more than once over time)	Repeated exposure assessment (more than once over time)	Blinding of outcome assessors	Follow-up rate	Statistical analysis	Quality rating percentage	Quality rating score (Good (67–100%), Fair (33–66%), Poor (0–33%))
Yan at al., 2015	Yes	Yes	Yes	Yes	No	Yes	Yes	Yes	Yes	Yes	No	NA	Yes	Yes	79%	Good
Yingfen et al.,2020	Yes	Yes	Yes	Yes	No	Yes	Yes	Yes	Yes	Yes	NA	NA	NA	Yes	71%	Good
Tang et al.,2020	Yes	Yes	Yes	Yes	No	Yes	Yes	Yes	Yes	Yes	Yes	NA	Yes	Yes	86%	Good
chan et al.,2015	Yes	Yes	Yes	Yes	No	Yes	Yes	NA	Yes	Yes	Yes	NA	Yes	Yes	79%	Good
Jiang et al., 2020	Yes	Yes	Yes	Yes	No	Yes	Yes	Yes	Yes	Yes	No	NA	Yes	Yes	79%	Good
Dong et al., 2020	Yes	Yes	Yes	Yes	No	Yes	Yes	Yes	Yes	Yes	No	NA	Yes	Yes	79%	Good
liu et al., 2019	Yes	Yes	Yes	Yes	No	Yes	Yes	Yes	Yes	Yes	No	NA	Yes	Yes	79%	Good
Lu et al., 2020	Yes	Yes	Yes	Yes	No	Yes	Yes	Yes	Yes	Yes	Yes	NA	Yes	Yes	86%	Good
Mo et ., 2018	Yes	Yes	Yes	Yes	No	Yes	Yes	Yes	Yes	Yes	No	NA	Yes	Yes	79%	Good
Kang etal., 2018	Yes	Yes	Yes	Yes	No	Yes	Yes	Yes	Yes	Yes	Yes	NA	Yes	Yes	86%	Good
Wu et al., 2020	Yes	Yes	Yes	Yes	No	Yes	CD	CD	Yes	Yes	No	NA	Yes	Yes	64%	Fair

CD, cannot determine; NA, not applicable.
