# Supplementary material for: Prognostic Epstein-Barr Virus (EBV) miRNA biomarkers for survival outcome in EBV-associated epithelial malignancies: Systematic review and meta-analysis
Source: PLoS One. 2022 Apr 18;17(4):e0266893. doi: 10.1371/journal.pone.0266893 (PMC9015129; doi:10.1371/journal.pone.0266893)
Supplement: S1 Fig — The funnel plot depicts the precision of the study size and standard error on the vertical axis as a function of the effect size on the horizontal axis. The individual study is represented by a dot, and most of this area comprises regions of high significance, indicating that publication bias would be reflected by asymmetrical distribution of dots. The smaller studies were represented at the bottom of the funnel plot at the bottom of the, where these studies are more likely to be published if they have larger-than-average effects, making them more likely to achieve the statistical significance criterion. (PDF) [file pone.0266893.s003.pdf]

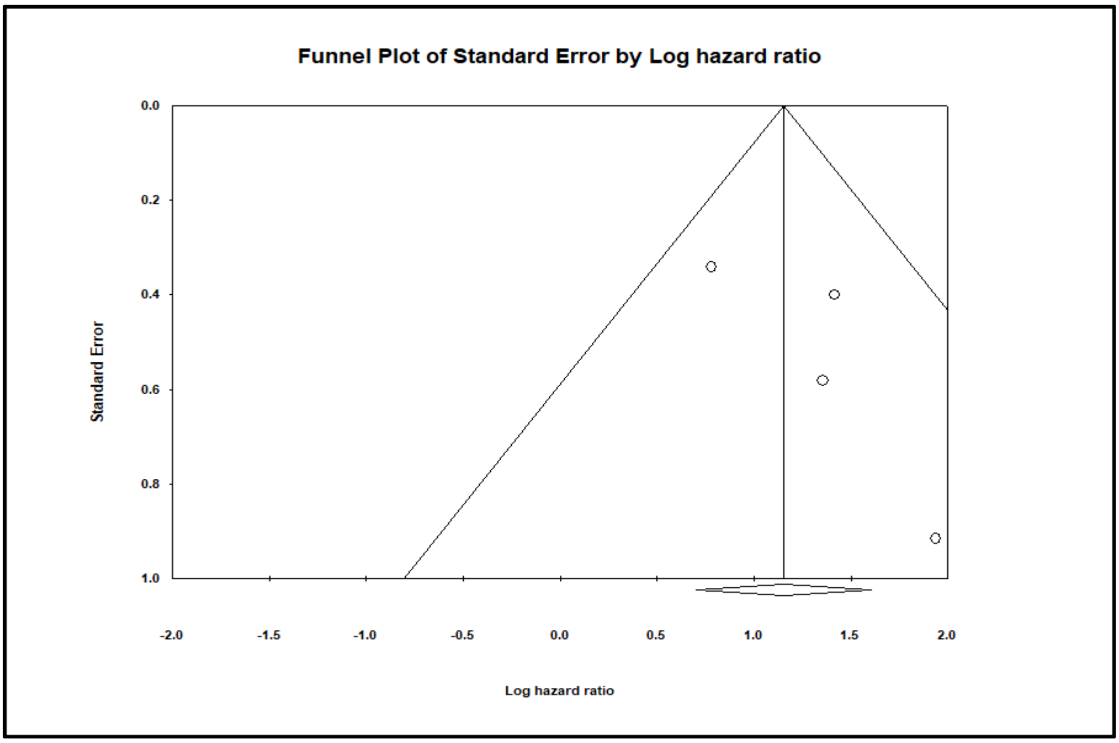

S1 Fig. Funnel plot of Standard Error by Log hazard ratio associating NPC and GC patients' survival with EBV microRNA expression. The funnel plot depicts the precision of the study size and standard error on the vertical axis as a function of the effect size on the horizontal axis. The individual study is represented by a dot, and most of this area comprises regions of high significance, indicating that publication bias would be reflected by asymmetrical distribution of dots The smaller studies were represented at the bottom of the funnel plot at the bottom of the, where these studies are more likely to be published if they have larger-than-average effects, making them more likely to achieve the statistical significance criterion.
