# Supplementary material for: Prognostic Epstein-Barr Virus (EBV) miRNA biomarkers for survival outcome in EBV-associated epithelial malignancies: Systematic review and meta-analysis
Source: PLoS One. 2022 Apr 18;17(4):e0266893. doi: 10.1371/journal.pone.0266893 (PMC9015129; doi:10.1371/journal.pone.0266893)
Supplement: S2 Fig — The Smaller studies are clustered at the bottom of the graph, and they will be distributed throughout a wide range of values because of the greater sampling variation in effect size estimates in the smaller studies. The funnel plot displays the precision and standard error of the study size on the vertical axis as a measure of the effect size on the horizontal axis. Individual studies are displayed by dots, and most of this area comprises regions of high significance, showing that publication bias is shown as asymmetrical graph. If smaller studies (those that appear at the bottom of the funnel plot) exhibit larger-than-average effects, they are more likely to be published, and they are more likely to achieve the statistical significance criterion, as would be expected. (PDF) [file pone.0266893.s004.pdf]

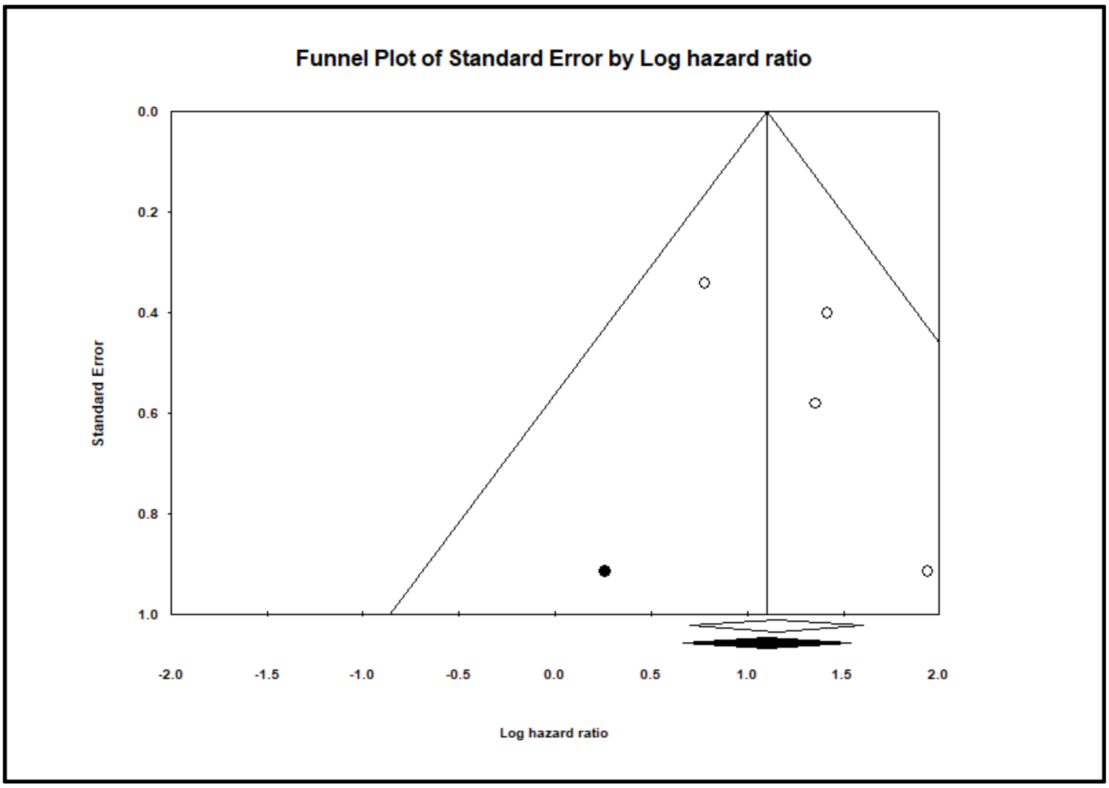

S2 Fig. Funnel plot containing observed and adjusted studies. The Smaller studies are clustered at the bottom of the graph, and they will be distributed throughout a wide range of values because of the greater sampling variation in effect size estimates in the smaller studies. The funnel plot displays the precision and standard error of the study size on the vertical axis as a measure of the effect size on the horizontal axis. Individual studies are displayed by dots, and most of this area comprises regions of high significance, showing that publication bias is shown as asymmetrical graph. If smaller studies (those that appear at the bottom of the funnel plot) exhibit larger-than-average effects, they are more likely to be published, and they are more likely to achieve the statistical significance criterion, as would be expected.
